# Alcoholic Stimulants in Typhus and Typhoid Fevers

**Published:** 1865-02

**Authors:** 


ARTICLE VI.

### ALCOHOLIC STIMULANTS IN TYPHUS & TYPHOID FEVERS.

BY THE EDITOR.

We call the particular attention of our readers to the interesting letter of our New York Correspondent, contained in the present number of the EXAMINER. The facts he gives, in regard to the mortality of typhus when placed in pure air, and under good diadetic regulations, without any medication or stimulants whatever, compared with the mortality of the same disease treated with active alcoholic medication, are of very great importance. The results, as given by our Correspondent, are in exact accordance with what we should have expected; and fully corroborates what we have persistently taught to our classes, both in the clinical wards and in the College lecture-room, during the last fifteen years. We have never been surprised, that under the profuse administration of alcoholic liquids, the mortality of typhus fever patients should be 1 in 6 in Bellevue, or 1 in 5 in the London fever hospitals. For if there is one single fact in pathology well established, it is, that the blood in typhus is imperfectly decarbonized, that its fibrine is impaired in coagulability, and that the functions of the nervous and muscular tissues are greatly depressed.

Equally well is the fact established, by a score of distinguished experimenters, that alcohol, while in the human system, diminishes the decarbonization of the blood, retards the coagulability of the fibrine, produces an anæsthetic or depressing effect on the nervous centres, and diminishes organic changes. Hence we could never perceive a rational indication for the use of alcohol in typhus or typhoid fevers; unless we call that homœopathic *humbug*, *Similia similibus curantur*, rational. We have attended the medical wards of the Mercy Hospital, in this City, for the last fifteen years. It is the only public hospital in the City, for the reception of fever patients, from the poorer classes of society; and during the period mentioned, there have been received and treated in its wards several hundred cases of typhus and typhoid fevers. And if all the alcoholic liquors prescribed for internal administration, during their treatment, were collected together they would not fill a half-gallon measure.

The wards were often overcrowded, and though always clean, were by no means capable of extra ventilation.

And yet, if we exclude those cases brought to the Hospital in a moribund condition, and which died in from one to three days after admission, the ratio of mortality, for each year, has varied from 1 in 15, to 1 in 25.

While the facts given, in the letter of our Correspondent, abundantly prove that plenty of pure air and simple nourishment is greatly more successful in the treatment of typhus than impure air and alcoholic medication, they by no means prove that no kind of medication is beneficial in that disease. On the contrary, from our own observations, we are convinced that, if Dr. Loomis, in addition to pure air and proper nourishment, had given his patients, judiciously, such medicines as are calculated to counteract septic or deteriorating changes in the blood; to lessen the tendency to irritation and disintegration of the mucous membrane of the ilium; and to increase the sensibility and action of the nervous centres in the more depressed cases, he would have reduced the mortality not only to 1 in 17, but to less than 1 in 20.

